# Whole genome sequencing insight into carbapenem-resistant and multidrug-resistant *Acinetobacter baumannii* harboring chromosome-borne *bla*_OXA-23_

**DOI:** 10.1128/spectrum.00501-24

**Published:** 2024-08-05

**Authors:** Wei Wang, Jiahui Weng, Jie Wei, Qinghuan Zhang, Yu Zhou, Yanju He, Limei Zhang, Wenting Li, Yi Zhang, Zhiren Zhang, Xiaobin Li

**Affiliations:** 1 Department of Pulmonary and Critical Care Medicine, Zhuhai People’s Hospital (Zhuhai Clinical Medical College of Jinan University), Zhuhai, China; 2 Department of Critical Care Medicine, Zhuhai People’s Hospital (Zhuhai Clinical Medical College of Jinan University), Zhuhai, China; 3 School of Basic Medical Sciences, Guangzhou University of Chinese Medicine, Guangzhou, China; 4 Department of Clinical Laboratory, Zhuhai People’s Hospital (Zhuhai Clinical Medical College of Jinan University), Zhuhai, China; 5 Department of Anesthesiology, Zhuhai People’s Hospital (Zhuhai Clinical Medical College of Jinan University), Zhuhai, China; 6 Guangdong Provincial Key Laboratory of Tumor Interventional Diagnosis and Treatment, Zhuhai People’s Hospital (Zhuhai Clinical Medical College of Jinan University), Zhuhai, China; MultiCare Health System, Tacoma, Washington, USA

**Keywords:** carbapenem-resistant *Acinetobacter baumannii*, chromosome-borne *bla*_OXA-23_, ST, genetic context, comparative genomic analysis

## Abstract

Carbapenem-resistant *Acinetobacter baumannii* (CRAB) poses a significant threat to hospitalized patients as effective therapeutic options are scarce. Based on the genomic characteristics of the CRAB strain AB2877 harboring chromosome-borne *bla*_OXA-23_, which was isolated from the bronchoalveolar lavage fluid (BALF) of a patient in a respiratory intensive care unit (RICU), we systematically analyzed antibiotic resistance genes (ARGs) and the genetic context associated with ARGs carried by CRAB strains harboring chromosome-borne *bla*_OXA-23_ worldwide. Besides *bla*_OXA-23_, other ARGs were detected on the chromosome of the CRAB strain AB2877 belonging to ST208/1806 (Oxford MLST scheme). Several key genetic contexts associated with the ARGs were identified on the chromosome of the CRAB strain AB2877, including (1) the MDR region associated with *bla*_OXA-23_, *tet(B)-tetR(B*), *aph(3'')-Ib,* and *aph(6)-Id* (2); the resistance island AbGRI3 harboring *armA* and *mph(E)-msr(E*) (3); the Tn*3*-like composite transposon containing *bla*_TEM-1D_ and *aph(3')-Ia*; and (4) the structure “IS*Aba1-bla*_ADC-25_.” The first two genetic contexts were most common in ST195/1816, followed by ST208/1806. The last two genetic contexts were found most frequently in ST208/1806, followed by ST195/1816.

**IMPORTANCE:**

The *bla*_OXA-23_ gene can be carried by plasmid or chromosome, facilitating horizontal genetic transfer and increasing carbapenem resistance in healthcare settings. In this study, we focused on the genomic characteristics of CRAB strains harboring the chromosome-borne *bla*_OXA-23_ gene, and the important genetic contexts associated with *bla*_OXA-23_ and other ARGs were identified, and their prevalent clones worldwide were determined. Notably, although the predominant clonal CRAB lineages worldwide containing the MDR region associated with *bla*_OXA-23_, *tet(B)-tetR(B*), *aph(3'')-Ib,* and *aph (6)-Id* was ST195/1816, followed by ST208/1806, the CRAB strain AB2877 in our study belonged to ST208/1806. Our findings contribute to the knowledge regarding the dissemination of CRAB strains and the control of nosocomial infection.

## INTRODUCTION

*Acinetobacter baumannii*, a ubiquitous, strictly aerobic, non-fermentative Gram-negative bacillus, has become an important opportunistic pathogen in nosocomial infections, which commonly occur in patients with high-risk factors, including immunocompromised status, old age, heavy use of antibiotics, indwelling catheters, and length of hospital and/or intensive care unit (ICU) stay (1, 2). The mortality rate of nosocomial blood infections caused by *A. baumannii* has reached 34% and up to 43% of blood infections in patients in the ICU (3). The success of *A. baumannii* as a nosocomial pathogen is likely attributable to its high antibiotic resistance (4).

The emergence of multidrug-resistant (MDR) and extremely drug-resistant (XDR) *A. baumannii* has made it one of the most troublesome nosocomial pathogens worldwide in recent years (5). Data from the China Antimicrobial Surveillance Network (CHINET) indicate that the resistance levels of *A. baumannii* isolates to imipenem and meropenem will remain relatively stable (accounting for more than 75%) from 2018 to 2022 (6). Indeed, in 2017, the World Health Organization listed CRAB as a priority one critical pathogen, with an urgent need for the development of new treatments (7).

Horizontal gene transfer and mutational changes in chromosomal structures are the main causes of multidrug-resistant (MDR) and extreme drug-resistant (XDR) *A. baumannii* (8). The spread of antibiotic resistance genes (ARGs) is facilitated by mobile genetic elements, such as plasmids, insertion sequences (ISs), transposons, and integrons (9). To better understand the characteristics of ARGs and the genetic environment associated with ARGs carried by the *A. baumannii* strain AB2877*,* which was isolated from a patient in a tertiary hospital, we performed *in silico* typing and comparative analysis of *the A. baumannii* strain AB2877 with other *A. baumannii* strains available in the NCBI database. This study highlights the important relationship between ARGs and mobile genetic elements in *A. baumannii* that harbor chromosome-borne *bla*_OXA-23_.

## RESULTS

### Antibiotic resistance profiles of *A. baumannii* strain AB2877

Antimicrobial susceptibility testing showed that the *A. baumannii* strain AB2877 was resistant to cephalosporins (ceftazidime and cefepime), carbapenems (imipenem and meropenem), quinolones (ciprofloxacin and levofloxacin), aminoglycosides (amikacin and tobramycin) and β-lactam/β-lactamase inhibitor combinations (piperacillin/tazobactam, ampicillin/sulbactam, cefoperazone/sulbactam, and ticarcillin/clavulanate) (Table 1). It was also resistant to doxycycline and sulfamethoxazole (Table 1). Additionally, the *A. baumannii* strain AB2877 showed intermediate-level resistance to tigecycline and minocycline (Table 1). Notably, for the antibiotics tested, the *A. baumannii* strain AB2877 was only susceptible to colistin and cefiderocol (Table 1).

**TABLE 1:** Minimum inhibitory concentration (MIC) values of CRAB AB2877^*b*^

Antibiotics		MIC(μg/mL)	R or S
Categories	Name		
Cephalosporins	Ceftazidime	≥ 64	R
	Cefepime	≥ 32	R
	Cefiderocol^*a*^	-	S
Carbapenems	Imipenem	≥ 16	R
	Meropenem	≥ 16	R
Quinolones	Ciprofloxacin	≥ 4	R
	Levofloxacin	≥ 8	R
Tetracyclines	Doxycycline	≥ 16	R
	Minocycline	8	I
	Tigecycline	4	I
Aminoglycosides	Amikacin	≥ 64	R
	Tobramycin	≥ 16	R
Sulfonamides	Sulfamethoxazole	≥ 320	R
Polymyxins	Colistin	≤ 0.5	S
β-lactam/β-lactamase inhibitor combinations	Piperacillin/tazobactam	≥ 128/4	R
	Ampicillin/sulbactam	≥ 32/16	
	Cefoperazone/sulbactam	≥ 64/32	R
	Ticarcillin/clavulanate	≥ 128/2	R

^*a*^ Cefiderocol, KB = 24.

^*b*^ S, Susceptible; R, Resistant; I, Intermediate.

### Genomic analysis of the *A. baumannii* strain AB2877

Genome analysis revealed that the *A. baumannii* strain AB2877 comprised a 3.89 Mb chromosome and a plasmid (pAB2877) of 8731 bp size. MLST analysis revealed that the *A. baumannii* strain AB2877 belonged to ST208/1806 (Oxford MLST scheme) and ST2 (Pasteur MLST scheme). The chromosome of the *A. baumannii* strain AB2877 harbored 13 acquired ARGs, including beta-lactam resistance genes (*bla*_ADC-73_, *bla*_OXA-23_, *bla*_OXA-66_, *bla*_TEM-1D_), sulphonamide resistance gene (*sul*2), tetracycline resistance (*tet(B*) and *tet(R*)), aminoglycoside resistance (*aph(3')-Ia*, *aph(3'')-Ib*, *aph (6)-Id*, and *arm*A), and macrolide resistance (*mph(E*) and *msr(E*)). The genome also contains several insertion sequence (IS) elements, most of which belong to the IS*4*, IS*5*, IS*6*, IS*66*, and IS*91* families. However, no ARGs or virulence genes were found in plasmid pAB2877. Notably, ARGs carried by the chromosome were categorized into five multi-drug resistant (MDR) regions.

### MDR region associated with *bla*_OXA-23_, *tet(B)-tetR(B*), *aph(3'')-Ib,* and *aph(6)-Id*

**Fig 1:**
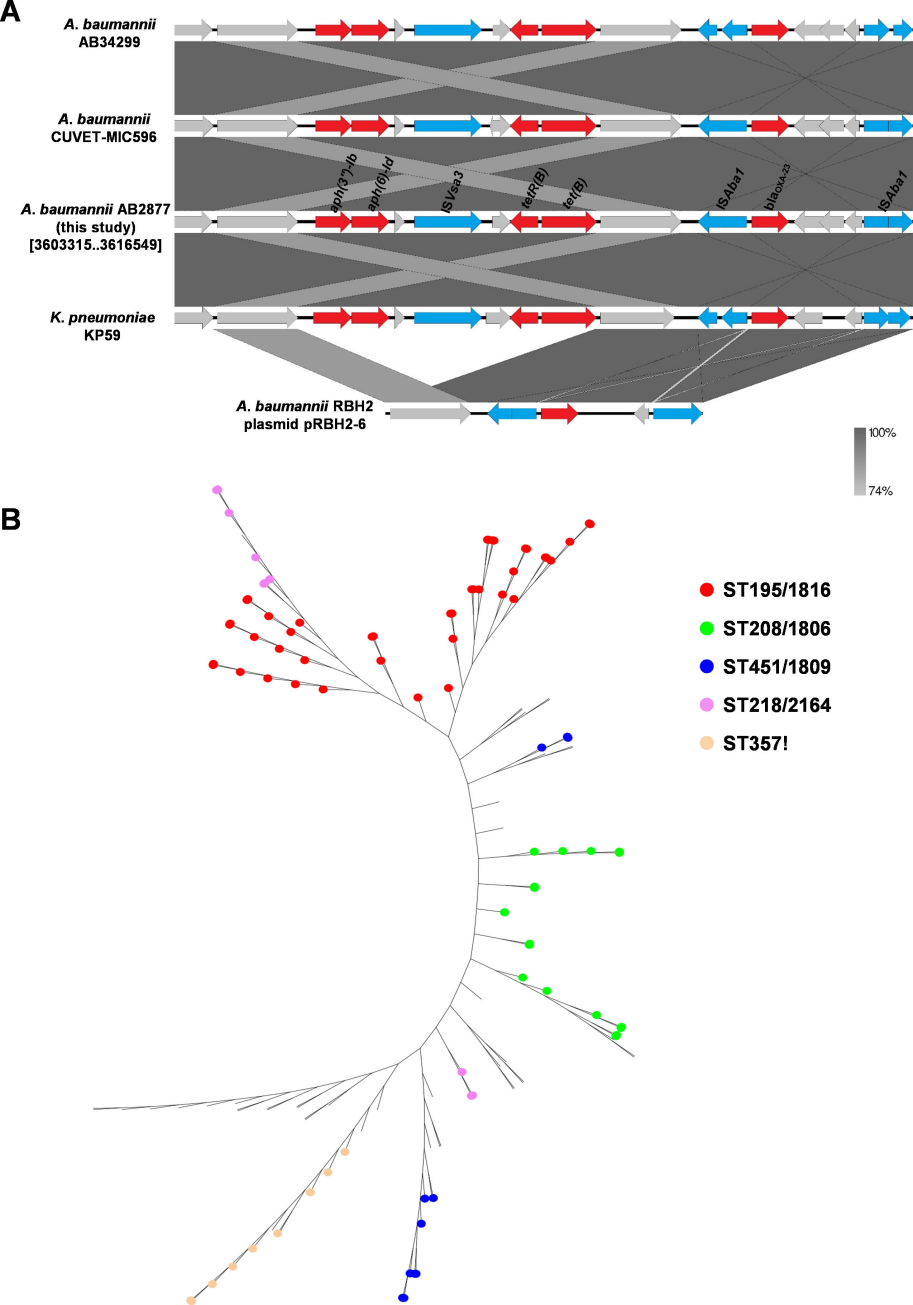
(**A**) Genetic structures of the MDR region associated with *bla_OXA-23_*, *tet(B*), *tetR(B*), *aph(3'')-Ib,* and *aph (6)-Id*. Genome sequences used to draw the diagrams from GenBank (*A. baumannii* VB31459 plasmid, *A. baumannii* AB34299 chromosome, *A. baumannii* CUVET-MIC596 chromosome, *A. baumannii* AB2877 chromosome, *K. pneumoniae* KP59 chromosome, *A. baumannii* RBH2 plasmid, *A. baumannii* VB82 plasmid). Genes and ORFs are indicated by arrows, and the direction of transcription is indicated by arrowheads. The resistance and transposase genes are shown in red and blue, respectively. (**B**) The unrooted tree shows the Top 5 most prevalent MLST from 135 strains of *A. baumannii*. The phylogenetic tree was created using the kSNP v3.1 based on the whole genomes of 135 strains of *A. baumannii*, which harbored the MDR region associated with *bla_OXA-23_*, *tet(B*), *tetR(B*), *aph(3'')-Ib,* and *aph (6)-Id*. The five most common MLST genotypes are ST195/1816 (red), ST208/1806 (green), ST451/1809 (blue), ST218/2164 (purple), and ST357! (orange).

The genes encoding beta-lactam resistance (*bla*_OXA-23_), tetracycline resistance (*tet(B)-tetR(B*)), and aminoglycoside resistance (*aph(3'')-Ib* and *aph (6)-Id*) were located in the ~13 kb MDR region (Fig. 1A). The *bla*_OXA-23_ gene, together with the DEAD/DEAH box helicase-like and ATPase genes, were flanked by two copies of *ISAba1* in different orientations, thus constituting the composite transposon Tn*2006* (Fig. 1A). In this MDR region, the insertion sequence IS*Vsa3* is located upstream and downstream of the aminoglycoside (*aph(3'')-Ib* and *aph (6)-Id*) and the tetracycline (*tet(B)-tetR(B*)) resistance genes, respectively (Fig. 1A).

BLAST analysis using the GenBank nr database based on the 13-kb MDR region carried by the *A. baumannii* strain AB2877 was conducted, and the results indicated that the most common species carrying the 13-kb MDR region was *A. baumannii* (135 fully sequenced genomes of *A. baumannii*; coverage ≥99% and identity ≥99%). In addition, we found that the 13-kb MDR region was also present on the chromosome of *Klebsiella pneumoniae* (GenBank accession CP076322, 100.00% coverage with 99.97% identity; Fig. 1A). Of the 135 *A*. *baumannii* strains encoding the 13-kb MDR region, the Top 5 prevalent Oxford STs (in descending order) were ST195/1816 (42 strains), ST208/1806 (17 strains), ST451/1809 (11 strains), ST218/2164 (10 strains), and ST357! (nine strains) (Fig. 1B).

### Genetic context associated with *armA* and *mph(E)-msr(E*)

**Fig 2:**
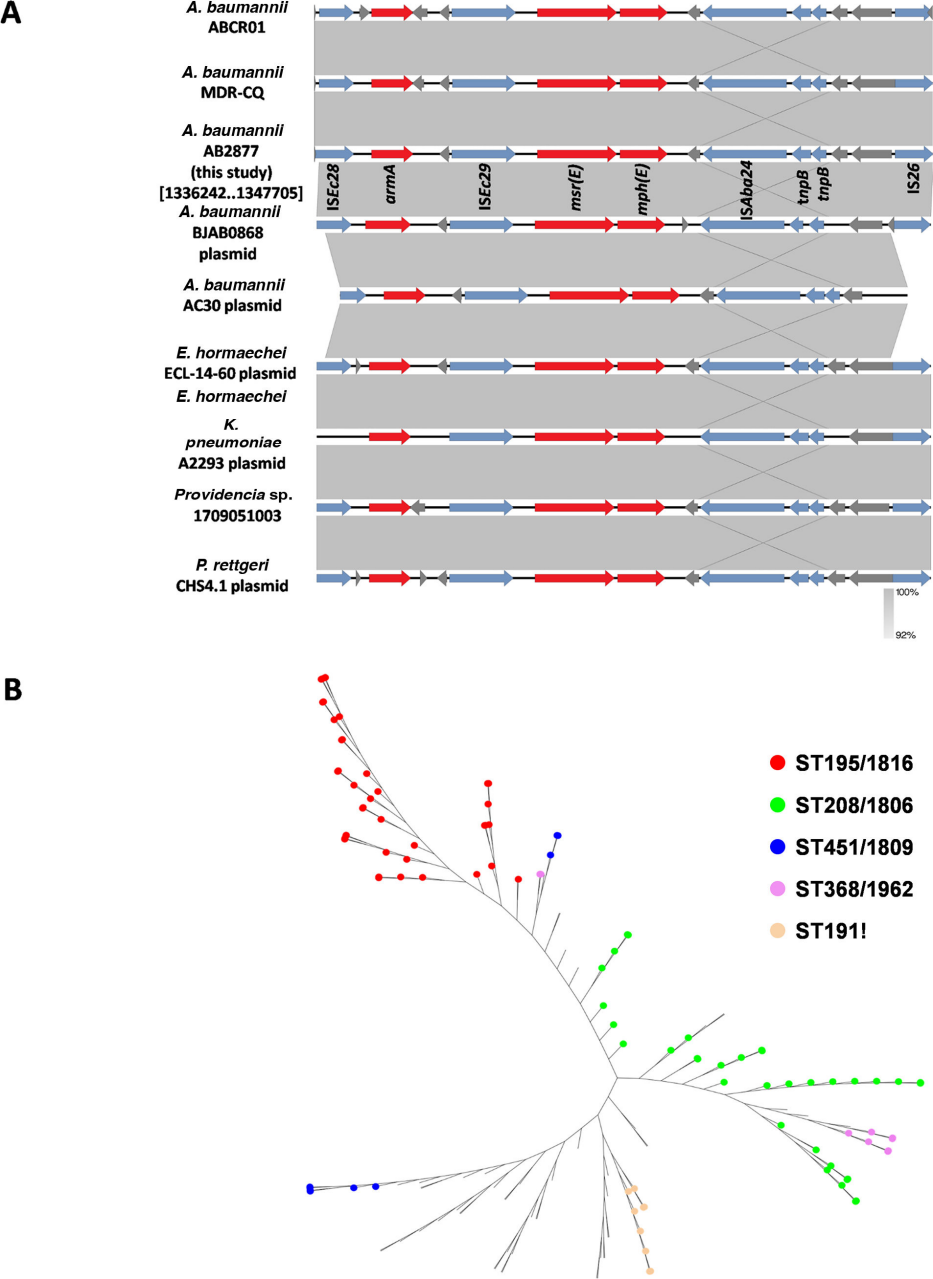
(**A**) Genetic contexts associated with *armA* and *mph(E)-msr(E*). The genome sequences used to draw the diagrams from GenBank (*A. baumannii* ABCR01 chromosome, *A. baumannii* MDR-CQ chromosome, *A. baumannii* AB2877 chromosome, *A. baumannii* BJAB0868 plasmid, *A. baumannii* AC30 plasmid, *E. hormaechei* ECL-14–60 plasmid, *K. pneumoniae* A2293 plasmid, *Providencia* sp. 1709051003 chromosome, and *P*. *rettgeri* CHS4.1 plasmid). Genes and ORFs are shown as arrows and the direction of transcription is indicated by arrowheads. The resistance and transposase genes are shown in red and blue, respectively. (**B**) The unrooted tree shows the TOP5 most prevalent MLST from 154 strains of *A. baumannii*. The phylogenetic tree was created using the kSNP v3.1 based on the whole genomes of 154 strains of *A. baumannii*, which harbored the genetic contexts associated with *armA* and *mph(E)-msr(E*). The five most common MLST genotypes are ST195/1816 (red), ST208/1806 (green), ST451/1809 (blue), ST368/1962 (purple), and ST191! (orange).

*armA* and *mph(E)-msr(E*) were located on a ~11.5-kb Tn*6180*-derived fragment of the resistance island AbGRI3, which was bracketed by IS*Ec28* and IS*26* (Fig. 2A). IS*Ec28* was inserted upstream of the *armA* gene. For the macrolide resistance genes (*mph(E)-msr(E*)), an IS*4*-like element IS*Ec29* was inserted upstream of the *mph(E)-msr(E*), and an IS*66*-like *element* IS*Aba24* was inserted downstream of the *mph(E)-msr(E)* (Fig. 2A).

Based on the BLAST analysis using the GenBank nr database, with a minimum coverage of 99% and minimum identity of 99%, the resistance island AbGRI3 harboring *armA* and *mph(E)-msr(E*) carried by the *A. baumannii* strain AB2877 was present not only in the chromosomes of *A. baumannii* (154 fully sequenced genomes of *A. baumannii*) but also in *A. baumannii* (*e.g. A. baumannii* BJAB0868 plasmid p3BJAB0868). The resistance island AbGRI3 harboring *armA* and *mph(E)-msr(E*) carried by the *A. baumannii* strain AB2877 was also identified in other species, including *Enterobacter hormaechei* (plasmid, GenBank accession MZ836805), *K. pneumoniae* (plasmid, GenBank accession MN310378), *Providencia* (both chromosomes [GenBank accession CP042861] and plasmid [GenBank accession OL908906]). Of the 154 *A*. *baumannii* strains that carried the 11.5-kb resistance island AbGRI3 harboring *armA* and *mph(E)-msr(E*), the dominant Oxford STs of TOP5 (in descending order) were ST195/1816 (39 strains), ST208/1806 (34 strains), ST451/1809 (9 strains), ST368/1962 (9 strains), and ST191! (nine strains) (Fig. 2B).

### Genetic context associated with *bla*_TEM-1D_ and *aph(3')-Ia*

**Fig 3:**
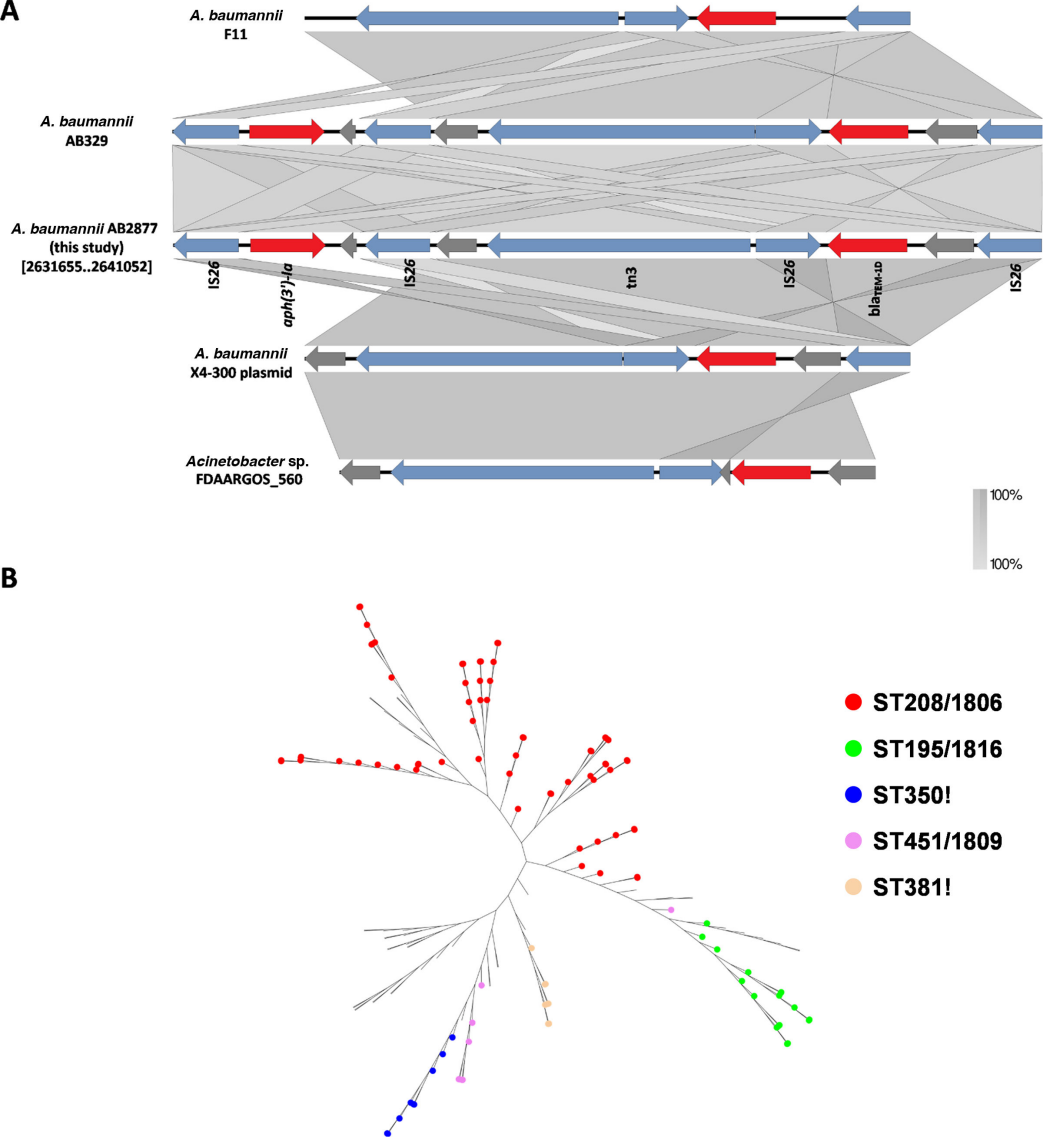
(**A**) Genetic structures associated with *bla*_TEM-1D_ and *aph(3')-Ia*. Genome sequences used to draw the diagrams from GenBank (*A. baumannii* F11, *A. baumaannii* AB329 chromosome, *A. baumannii* AB2877 chromosome, *A. baumannii* X4-300 plasmid, and *Acinetobacter* sp. FDAARGOS_560 chromosome). Genes and ORFs are shown as arrows, and the direction of transcription is indicated by arrowheads. The resistance and transposase genes are shown in red and blue, respectively. (**B**) The unrooted tree shows the Top 5 most prevalent MLST from 156 strains of *A. baumannii*. The phylogenetic tree was created using the kSNP v3.1 based on the whole genomes of 156 strains of *A. baumannii*, which harbored the genetic structures associated with *bla*_TEM-1D_ and *aph(3')-Ia*. The five most common MLST genotypes are ST208/1806 (red), ST195/1816 (green), ST350! (blue), ST451/1809 (purple), and ST381! (orange).

*bla*_TEM-1D_ and *aph(3')-Ia* were located on a 9.4-kb Tn*3*-like composite transposon, which was bracketed by two copies of IS*26* in the same orientation (Fig. 3A). Additionally, four copies of IS*26* in the same orientation were found in the 9.4-kb Tn*3*-like composite transposon. The *bla*_TEM-1D_ gene was flanked by two direct repeats of IS*26*, and the *aph(3')-Ia* gene was also flanked by two direct repeats of IS*26* (Fig. 3A).

Based on the BLAST analysis hit from the GenBank nr database, with a minimum coverage of 99% and a minimum identity of 99%, the 9.4-kb Tn*3*-like composite transposon containing *bla*_TEM-1D_ and *aph(3')-Ia* was widely present on the chromosomes of *A. baumannii* (156 fully sequenced genomes of *A. baumannii*; coverage ≥99% and identity ≥99%). The top 5 predominant Oxford STs among the 156 strains of *A. baumannii* (in descending order) were ST208/1806 (65 strains), ST195/1816 (17 strains), ST350! (nine strains), ST451/1809 (eight strains), and ST381! (eight strains) (Fig. 3B).

### Genetic context of *bla*_ADC-25_

**Fig 4:**
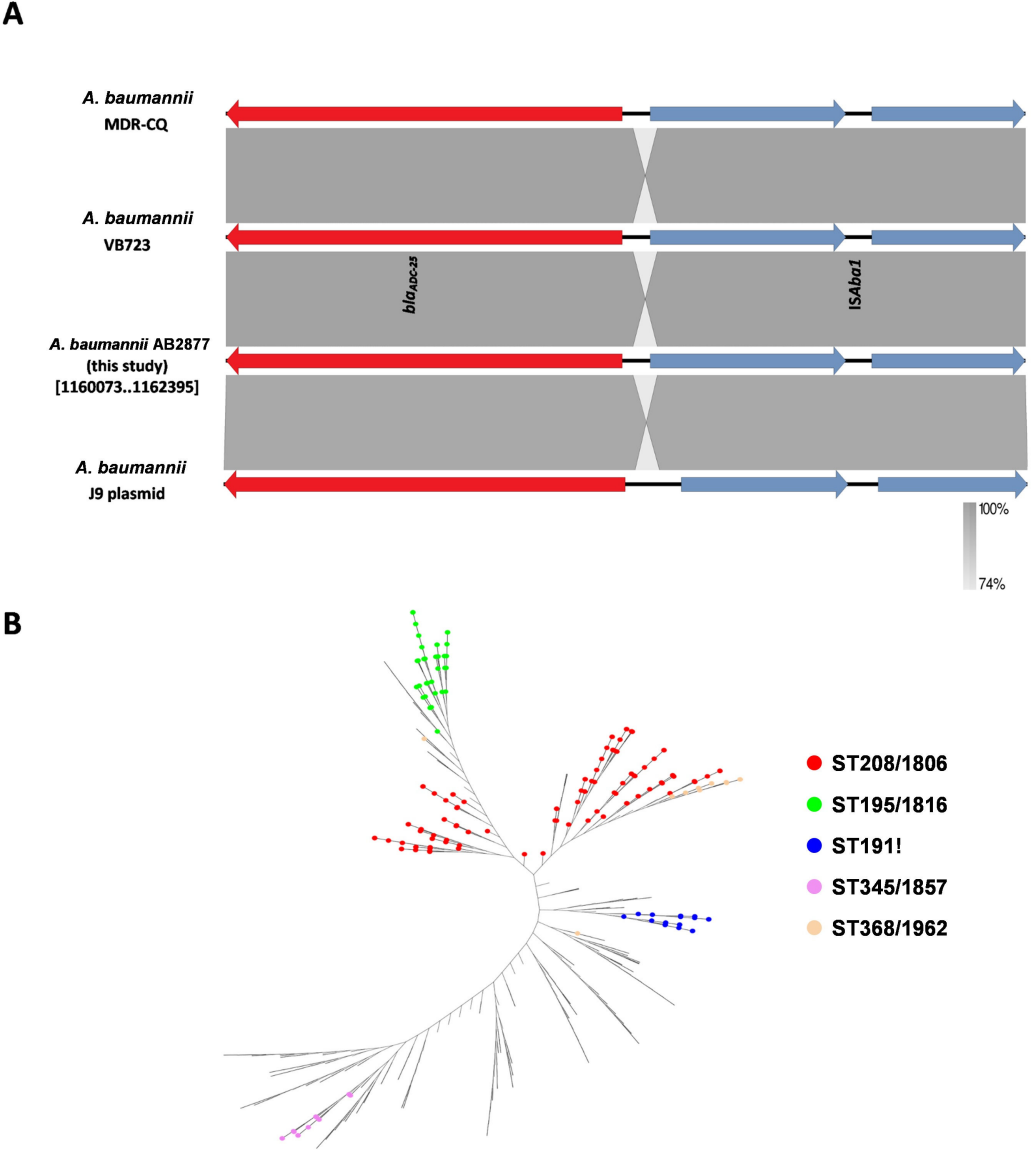
(**A**) Genetic structure of *bla*_ADC-25_. Genome sequences used to draw the diagrams from GenBank (*A. baumannii* MDR-CQ chromosome, *A. baumannii* VB723 chromosome, *A. baumannii* AB2877 chromosome, *A. baumannii* J9 plasmid). Genes and ORFs are shown as arrowheads, and the direction of transcription is indicated by arrowheads. The resistance and transposase genes are shown in red and blue, respectively. (**B**) The unrooted tree shows the most prevalent TOP5 MLST from 461 strains of *A. baumannii*. The phylogenetic tree was created using the kSNP v3.1 based on the whole genomes of 461 strains of *A. baumannii*, which harbored the genetic structure of *bla*_ADC-25_. The five most common MLST genotypes are ST208/1806 (red), ST195/1816 (green), and ST191! (blue), ST345/1857 (purple), and ST368/1962 (orange), respectively.

In the *A. baumannii* strain AB2877, IS*Aba1* was located downstream of *bla*_ADC-25_ (Fig. 4A). The structure “IS*Aba1-bla*_ADC-25_” was widely present on the chromosomes of *A. baumannii* (461 fully sequenced genomes of *A. baumannii*; coverage ≥99% and identity ≥99%). Of the 461 *A*. *baumannii* strains carrying the structure “IS*Aba1-bla*_ADC-25_,” the five most prevalent Oxford STs (in descending order) were ST208/1806 (114 strains), ST195/1816 (44 strains), ST191! (20 strains), ST345/1857 (16 strains), and ST368/1962(15 strains) (Fig. 4B).

## DISCUSSION

In this study, we describe a CRAB strain, AB2877, belonging to ST208/1806 (Oxford MLST scheme) or ST2 (Pasteur MLST scheme), which was isolated from the bronchoalveolar lavage fluid (BALF) of a 66-year-old patient in the respiratory intensive care unit (RICU). Among the antibiotics tested, the CRAB strain AB2877 was only susceptible to colistin and cefiderocol, indicating their great clinical value in the treatment of CRAB-causing infections. The CRAB strain AB2877 carries chromosomal *bla*_OXA-23_ gene, which is a key determinant of antibiotic resistance found in *Acinetobacter* species, which encodes a class D β-lactamase enzyme that confers resistance to carbapenem antibiotics (10). The *bla*_OXA-23_-like gene is prevalent in China and is present in 97% of carbapenem-resistant isolates (11). IS*Aba1* is a strong promoter sequence that influences the expression of neighboring genes, including those encoding antibiotic resistance determinants (12). Upstream of resistance genes, IS*Aba1*, can significantly increase their expression, leading to increased resistance to antibiotics (13).

In CRAB AB2877, the *bla*_OXA-23_ gene was embedded in Tn*2006*, which is bracketed with two copies of *ISAba1* in different orientations. The transposon Tn*2006* is one of the most common transposons worldwide (14) and may be one of the main reasons for the global dissemination of carbapenem resistance in *A. baumannii* (15). In particular, besides transposon Tn*2006*, transposons Tn2008 and Tn*2009* also appeared to contribute significantly to the dissemination of *bla*_OXA-23_ in China (16–18). Furthermore, we found that Tn*2006* harboring *bla*_OXA-23_ was present not only on the chromosomes but also on the plasmids of *A. baumannii*, as well as other pathogens, such as *K. pneumoniae* and *Proteus mirabilis* (19), suggesting that several species may be reservoirs or scatterers for this class D carbapenemase gene. In addition to the bla_OXA-23_ gene, other beta-lactamase genes (*bla*_OXA-66_, *bla*_ADC-25_, and *bla*_TEM-1D_) were also detected in this study. This finding is consistent with those of previous reports. For example, an *A. baumannii* isolate has been reported to harbor *bla*_OXA-23_, *blaOXA-66,* and *bla*_ADC-25_ in south China (20). Several coexisting multi-beta-lactam resistance genes contribute significantly to the extensive drug resistance observed in *A. baumannii* strains, which poses significant challenges for infection control and clinical management.

In this study, the CRAB strain AB2877 belonged to ST208/1806 (Oxford MLST scheme). Analysis of the genetic context associated with detected ARGs indicated that the genetic context associated with *bla*_TEM-1D_ and that of *bla*_ADC-25_ were easily detected in ST208/1806, followed by ST195/1816. The MDR regions associated with *bla*_OXA-23_, *tet(B)-tetR(B*), *aph(3'')-Ib*, *aph (6)-Id*, and the resistance island AbGRI3 harboring *armA* and *mph(E)-msr(E*) were commonly detected in ST195/1816, followed by ST208/1806. ST208 is widely recognized as a predominant lineage of *A. baumannii* GC2 worldwide, and some researchers believe that ST208 may have originated in North America and evolved into two clades (21). Reports of carbapenem-resistant *A. baumannii* ST195 in China have increased gradually in recent years (22, 23).

The *bla*_OXA-23_-like gene is almost always found within transposons and is commonly associated with an antibiotic-resistance genomic island (AbGRI) (14). The ribosomal RNA methyltransferase gene *armA*, an aminoglycoside resistance gene located on the resistance island AbGRI3, has been widely reported in *A. baumannii* (24). In the AB2877 isolate, the genetic contexts associated with *armA* and *mph(E)-msr(E*) were similar to those of Tn*6180*. Tn*6180*-borne *armA* or AbGRI3 has spread worldwide, especially in Japan and East Asia (25, 26).

In this study, we describe the genomic characteristics of the multidrug-resistant CRAB strain AB2877 belonging to ST208/1806 (Oxford MLST scheme) harboring chromosome-borne *bla*_OXA-23_, which was isolated from the BALF of a patient in the RICU in China. Several key genetic contexts associated with *bla*_OXA-23_ and other ARGs were found on the chromosome of the CRAB strain AB2877. Based on the genomes of *A. baumannii* available in the GenBank database, we explored the predominant clonal lineages of *A. baumannii* worldwide, including different genetic contexts of the CRAB strain AB2877. The *bla*_OXA-23_ gene was located in the MDR region associated with *bla*_OXA-23_, *tet(B)-tetR(B*), *aph(3'')-Ib,* and *aph (6)-Id*, which was most commonly found in ST195/1816, followed by ST208/1806. Furthermore, the resistance island AbGRI3 that harbors *armA* and *mph(E)-msr(E*) carried by strain AB2877 was most frequently found in CRAB ST195/1816, followed by CRAB ST208/1806. The CRAB strain AB2877 also carried one Tn*3*-like composite transposon bracketed by two copies of IS*26* containing *bla*_TEM-1D_ and *aph(3')-Ia* and one structure “IS*Aba1-bla*_ADC-25_,” which were most commonly found in ST208/1806, followed by ST195/1816.

## MATERIALS AND METHODS

### Isolation, identification, and antimicrobial susceptibility testing

The strain AB2877 was isolated from a BALF sample of a patient in the RICU who stayed for more than 14 days and received antibiotic treatment with carbapenem in the hospital. Bacterial species were identified using a fully automatic VITEK-2 Compact system (bioMérieux, France) and by sequencing the 16S rRNA gene. Antimicrobial susceptibility was measured using the VITEK-2 Compact system, which used the following antimicrobial agents: cephalosporins (ceftazidime, cefepime, and cefiderocol), carbapenems (imipenem and meropenem), quinolones (ciprofloxacin and levofloxacin), tetracyclines (doxycycline, minocycline, tigecycline), aminoglycosides (amikacin and tobramycin), sulfonamides (sulfamethoxazole), polymyxins (colistin), and β-lactam/β-lactamase inhibitor combinations (piperacillin/tazobactam, ampicillin/sulbactam, cefoperazone/sulbactam, and ticarcillin/clavulanate). The minimum inhibitory concentration (MIC) breakpoints for cefoperazone–sulbactam were those for *A. baumannii*: S, ≤16/8 mg/L; I, 32/16 mg/L; R, ≤64/32 mg/L. Colistin resistance was confirmed by broth microdilution test (Mikrolatest; Erba Lachema, Brno, Czech Republic) as suggested by EUCAST. Susceptibility testing for cefiderocol was determined by Kirby–Bauer’s disk diffusion (KB) method. The results of other antimicrobial agents were interpreted according to the Institute of Clinical and Laboratory Standards (CLSI M100–S33) (CLSI, 2023).

### Whole genome sequencing, assembly, and annotation

Whole-genome sequencing of the *A. baumannii* strain AB2877 was performed by GENEWIZ Co., Ltd. (Suzhou, China) using paired-end sequencing with Novaseq 6000 (150-bp paired-end reads) and long sequencing with PacBio Sequel. Hybrid assembly with long and short reads was used to produce the complete bacterial genome of the *A. baumannii* strain AB2877. First, PacBio long reads were assembled using HGAP (v.4.0)/Falcon (v.0.3) of WGS-Assembler 8.2 (27), and assembly polishing was performed with Pilon (version 1.22) (28) using Illumina short reads. The assembled genome of the *A. baumannii* strain AB2877 was submitted to the NCBI GenBank database (29) and annotated using the NCBI Prokaryotic Annotation Pipeline (PGAP) (30).

### Bioinformatics analysis for the genome of the *A. baumannii* strain AB2877

The multilocus sequence typing (MLST) of the *A. baumannii* strain AB2877 was performed using MLST software (v.2.0; https://cge.food.dtu.dk/services/MLST/) (31). ResFinder (32) v.4.5 (http://genepi.food.dtu.dk/resfinder) identified several acquired antimicrobial resistance genes. Insertion sequence (IS) elements were identified using ISfinder (https://www-is.biotoul.fr/blast.php) (33). A sequence similarity search was conducted against the GenBank non-redundant (nr) database using MegaBLAST (34). Sequence comparisons were visualized using Easyfig v.2.2.5 (35). Genome-wide single-nucleotide polymorphism (SNP) calling and phylogenetic analysis were performed by using kSNP v3.1 (36), and the tree was displayed with iTOL (37).

## Data Availability

The complete sequences of the chromosome and plasmid of *A. baumannii* strain AB2877 were submitted to GenBank database, under accession numbers CP092485-CP092486.
